# Crystal structure of ethyl (2*Z*)-2-cyano-3-[(3-methyl-1-phenyl-1*H*-pyrazol-5-yl)amino]­prop-2-enoate

**DOI:** 10.1107/S1600536814023502

**Published:** 2014-10-31

**Authors:** Joel T. Mague, Shaaban K. Mohamed, Mehmet Akkurt, Talaat I. El-Emary, Mustafa R. Albayati

**Affiliations:** a Department of Chemistry, Tulane University, New Orleans, LA 70118, USA; b Chemistry and Environmental Division, Manchester Metropolitan University, Manchester, M1 5GD, England; c Chemistry Department, Faculty of Science, Minia University, 61519 El-Minia, Egypt; d Department of Physics, Faculty of Sciences, Erciyes University, 38039 Kayseri, Turkey; e Department of Chemistry, Faculty of Science, Assiut University, 71515 Assiut, Egypt; f Kirkuk University, College of Science, Department of Chemistry, Kirkuk, Iraq

**Keywords:** crystal structure, pyrazole ring, disorder, acrylate compounds

## Abstract

The title compound, C_16_H_16_N_4_O_2_, crystallizes with two mol­ecules in the asymmetric unit, one of which shows disorder of the acetate group over two sets of sites in a 0.799 (2):0.201 (2) ratio. The phenyl group has a similar but opposite sense of twist relative to the pyrazole ring in the two mol­ecules, as indicated by the *syn* N—N—C_ar_—C_ar_ (ar = aromatic) torsion angles of 39.7 (2) and −36.9 (2)°. Each mol­ecule features an intra­molecular N—H⋯O hydrogen bond, which closes an *S*(6) ring. In the crystal, C—H⋯O and C—H⋯N inter­actions direct the packing into a layered structure parallel to (110).

## Related literature   

For the biological activities and industrial applications of acrylate compounds, see: Wang *et al.* (2003 ▶); Dillingham *et al.* (1983 ▶); Liu *et al.* (1999 ▶); Hsiao *et al.* (2004 ▶). For chemical versatility of the acrylate moiety, see: Kang & Fang (2004 ▶); Qiu *et al.* (2004 ▶).
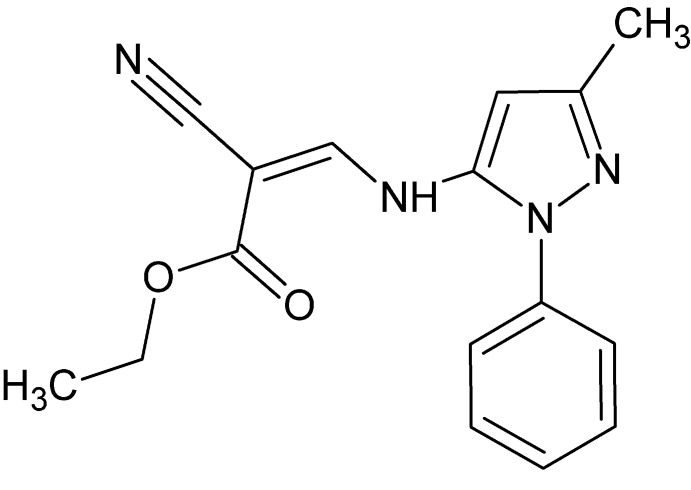


## Experimental   

### Crystal data  

C_16_H_16_N_4_O_2_
*M* *_r_* = 296.33
Triclinic, 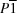

*a* = 9.0656 (2) Å
*b* = 10.4085 (3) Å
*c* = 16.5551 (4) Å
α = 86.9930 (11)°
β = 81.567 (1)°
γ = 73.3900 (11)°
*V* = 1480.67 (7) Å^3^
*Z* = 4
Cu *K*α radiation
μ = 0.74 mm^−1^
*T* = 150 K
0.20 × 0.12 × 0.07 mm

### Data collection  

Bruker D8 VENTURE PHOTON 100 CMOS diffractometer
Absorption correction: multi-scan (*SADABS*; Bruker, 2014 ▶) *T*_min_ = 0.87, *T*_max_ = 0.95
19446 measured reflections
5749 independent reflections
4534 reflections with *I* > 2σ(*I*)
*R*_int_ = 0.033

### Refinement  

*R*[*F* ^2^ > 2σ(*F* ^2^)] = 0.042
*wR*(*F* ^2^) = 0.112
*S* = 1.05
5749 reflections
422 parameters
8 restraints
H-atom parameters constrained
Δρ_max_ = 0.23 e Å^−3^
Δρ_min_ = −0.22 e Å^−3^

### 

Data collection: *APEX2* (Bruker, 2014 ▶); cell refinement: *SAINT* (Bruker, 2014 ▶); data reduction: *SAINT*; program(s) used to solve structure: *SHELXT* (Sheldrick, 2008 ▶); program(s) used to refine structure: *SHELXL2014* (Sheldrick, 2008 ▶); molecular graphics: *DIAMOND* (Brandenburg & Putz, 2012 ▶); software used to prepare material for publication: *SHELXTL* (Sheldrick, 2008 ▶).

## Supplementary Material

Crystal structure: contains datablock(s) global, I. DOI: 10.1107/S1600536814023502/hb7306sup1.cif

Structure factors: contains datablock(s) I. DOI: 10.1107/S1600536814023502/hb7306Isup2.hkl

Supporting information file. DOI: 10.1107/S1600536814023502/hb7306Isup3.cml

**Fig. 1.**
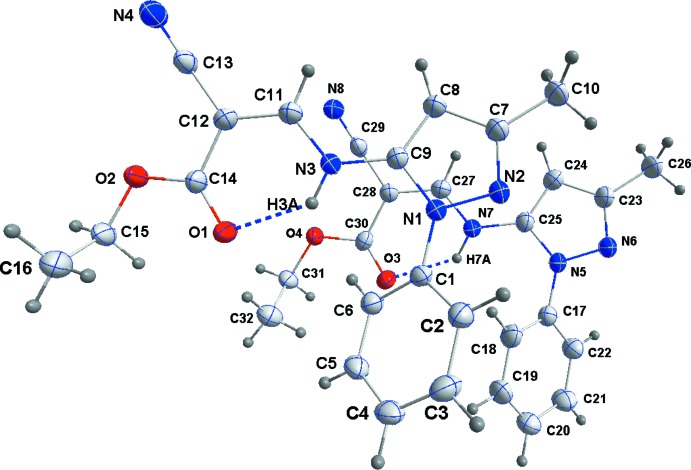
Perspective view of the asymmetric unit with 50% probability ellipsoids and intramolecular N—H···O hydrogen bonds shown as dotted lines. Only the major portion of the disorder in molecule 1 is shown.

. DOI: 10.1107/S1600536814023502/hb7306fig1.tif
Perspective view of the asymmetric unit with 50% probability ellipsoids and intra­molecular N—H⋯O hydrogen bonds shown as dotted lines. Only the major portion of the disorder in mol­ecule 1 is shown.

**Fig. 2.**
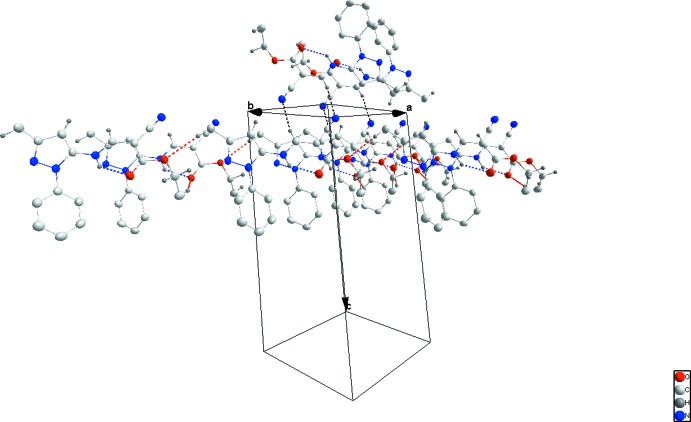
Packing viewed towards the [110] plane with intramolecular N—H···O hydrogen bonds shown as blue dotted lines and intermolecular C—H···O and C—H···N interactions as red and black dotted lines, respectively.

. DOI: 10.1107/S1600536814023502/hb7306fig2.tif
Packing viewed towards the [110] plane with intra­molecular N—H⋯O hydrogen bonds shown as blue dotted lines and inter­molecular C—H⋯O and C—H⋯N inter­actions as red and black dotted lines, respectively.

**Fig. 3.**
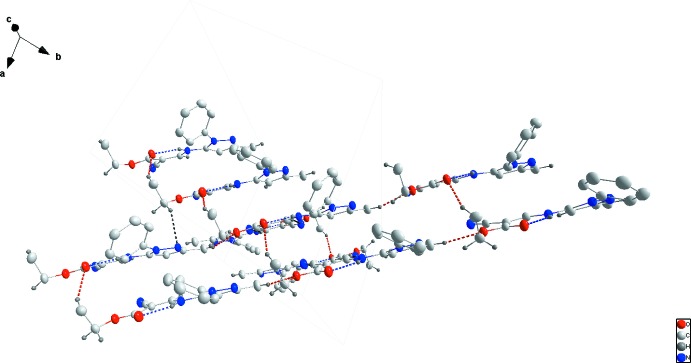
Packing showing the layer structure with intramolecular N—H···O hydrogen bonds shown as blue dotted lines and intermolecular C—H···O and C—H···N interactions as red and black dotted lines, respectively.

. DOI: 10.1107/S1600536814023502/hb7306fig3.tif
Packing showing the layer structure with intra­molecular N—H⋯O hydrogen bonds shown as blue dotted lines and inter­molecular C—H⋯O and C—H⋯N inter­actions as red and black dotted lines, respectively.

CCDC reference: 1031059

Additional supporting information:  crystallographic information; 3D view; checkCIF report

## Figures and Tables

**Table 1:** Hydrogen-bond geometry (, )

*D*H*A*	*D*H	H*A*	*D* *A*	*D*H*A*
C15H15*B*N2^i^	0.99	2.63	3.455(2)	141
C16H16*C*O3^ii^	0.98	2.52	3.430(3)	155
N3H3*A*O1	0.91	1.96	2.677(4)	134
C10H10*C*O2^iii^	0.98	2.55	3.506(2)	164
C11H11N8^iv^	0.95	2.37	3.306(2)	168
N7H7*A*O3	0.91	2.00	2.7027(17)	133
C24H24N4^iv^	0.95	2.68	3.555(2)	153
C26H26*C*O4^iii^	0.98	2.55	3.523(2)	172
C27H27N4^iv^	0.95	2.40	3.322(2)	164
C31H31*A*N2^v^	0.99	2.57	3.366(2)	138
C31H31*B*N6^i^	0.99	2.63	3.437(2)	139
C32H32*C*O1	0.98	2.55	3.468(3)	155

Symmetry codes: (i) 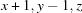; (ii) 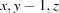; (iii) 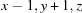; (iv) 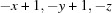; (v) 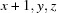.

## supplementary crystallographic information

### S1. Comment

Acrylate compounds have been receiving significant attention in the fields of materials and pharmaceutical sciences due to their physical and biological properties (Wang *et al.*, 2003; Dillingham *et al.*, 1983). For example, cyanoacrylates are widely used as inhibitors for the photosystem II (PSII) which inhibits the growth of weeds by disrupting photosynthetic electron transport (Liu *et al.*, 1999). Among these cyanoacrylates, 3-(4-chlorobenzyl)amino-2-cyano-3-isobutylacrylate exhibits the highest inhibitory activity of the Hill reaction (Wang *et al.*, 2003). Moreover, 3-aminoacrylates can also be hydrogenated into β-amino acid derivatives which have extensive application in life sciences as components of biologically active peptides and small-molecule pharmaceuticals (Hsiao *et al.*, 2004). In addition, acrylates also represent an important class of organic compounds which are employed as important intermediates in organic synthesis due to the chemical versatility of the acrylate moiety and continue to attract considerable attention of chemists (Kang & Fang, 2004; Qiu *et al.*, 2004). Based on such findings and following our on-going study of acrylate base pyrazoles we herein report the synthesis and crystal structure study of the title compound.

The title molecule crystallizes with two independent molecules in the asymmetric unit (Fig. 1). These differ primarily in the orientation of the phenyl ring with respect to the mean plane of the pyrazole ring. Thus the dihedral angle between the C1–C6 phenyl ring and the pyrazole ring built on N1 is 43.90 (6)° while that in the other molecule is 37.38 (6)°. The two molecules are nearly parallel as seen from the angle between the mean planes of the pyrazole cores of 2.5 (1)°. The molecular conformations are partly determined by intramolecular N3—H3a···O1 and N7—H7a···O3 hydrogen bonds (Table 2 and Fig. 1) while C—H···O and C—H···N interactions direct the packing into a layer structure (Fig. 3 and Table 2).

### S2. Experimental

A mixture of 3-methyl-1-phenyl-1H-pyrazol-5-amine 1.73 g (0.01 mol) and ethyl (2Z)-2-cyano-3-ethoxyacrylate 1.69 g (0.01 mol) in absolute ethanol (15 mL) was heated under reflux and monitored by TLC. On completion after 3 h, the reaction mixture was allowed to cool to ambient temperature. Solid yellow product was deposited, collected and dried under vacuum. Colourless crystals suitable for X-ray diffraction were obtained by recrystallisation of the product from ethanol. M.p. 448–450 K.

### S3. Refinement

H-atoms attached to carbon were placed in calculated positions (C—H = 0.95 - 0.98 Å) while those attached to nitrogen were placed in locations derived from a difference map and their parameters adjusted to give N—H = 0.91 Å. All were included as riding contributions with isotropic displacement parameters 1.2 - 1.5 times those of the attached atoms. The major portion of the side chain in molecule 1 is disordered over two reasonably resolved sites in a 4:1 ratio. The two components of the disorder were refined subject to restraints that their geometries be comparable to one another and to that of the corresponding ordered portion of molecule 2.

### Crystal data

**Table:**

C_16_H_16_N_4_O_2_	*Z* = 4
*M**_r_* = 296.33	*F*(000) = 624
Triclinic, *P*1	*D*_x_ = 1.329 Mg m^−^^3^
*a* = 9.0656 (2) Å	Cu *K*α radiation, λ = 1.54178 Å
*b* = 10.4085 (3) Å	Cell parameters from 9947 reflections
*c* = 16.5551 (4) Å	θ = 2.7–72.2°
α = 86.9930 (11)°	µ = 0.74 mm^−^^1^
β = 81.567 (1)°	*T* = 150 K
γ = 73.3900 (11)°	Column, colourless
*V* = 1480.67 (7) Å^3^	0.20 × 0.12 × 0.07 mm

### Data collection

**Table:**

Bruker D8 VENTURE PHOTON 100 CMOS diffractometer	5749 independent reflections
Radiation source: INCOATEC IµS micro–focus source	4534 reflections with *I* > 2σ(*I*)
Mirror monochromator	*R*_int_ = 0.033
Detector resolution: 10.4167 pixels mm^-1^	θ_max_ = 72.2°, θ_min_ = 2.7°
ω scans	*h* = −11→11
Absorption correction: multi-scan (*SADABS*; Bruker, 2014)	*k* = −11→12
*T*_min_ = 0.87, *T*_max_ = 0.95	*l* = −20→20
19446 measured reflections	

### Refinement

**Table:**

Refinement on *F*^2^	Primary atom site location: structure-invariant direct methods
Least-squares matrix: full	Secondary atom site location: difference Fourier map
*R*[*F*^2^ > 2σ(*F*^2^)] = 0.042	Hydrogen site location: mixed
*wR*(*F*^2^) = 0.112	H-atom parameters constrained
*S* = 1.05	*w* = 1/[σ^2^(*F*_o_^2^) + (0.0503*P*)^2^ + 0.4804*P*] where *P* = (*F*_o_^2^ + 2*F*_c_^2^)/3
5749 reflections	(Δ/σ)_max_ < 0.001
422 parameters	Δρ_max_ = 0.23 e Å^−^^3^
8 restraints	Δρ_min_ = −0.22 e Å^−^^3^

### Special details

**Table:**

Geometry. All e.s.d.'s (except the e.s.d. in the dihedral angle between two l.s. planes) are estimated using the full covariance matrix. The cell e.s.d.'s are taken into account individually in the estimation of e.s.d.'s in distances, angles and torsion angles; correlations between e.s.d.'s in cell parameters are only used when they are defined by crystal symmetry. An approximate (isotropic) treatment of cell e.s.d.'s is used for estimating e.s.d.'s involving l.s. planes.
Refinement. Refinement of *F*^2^ against ALL reflections. The weighted *R*-factor *wR* and goodness of fit *S* are based on *F*^2^, conventional *R*-factors *R* are based on *F*, with *F* set to zero for negative *F*^2^. The threshold expression of *F*^2^ > σ(*F*^2^) is used only for calculating *R*-factors(gt) *etc*. and is not relevant to the choice of reflections for refinement. *R*-factors based on *F*^2^ are statistically about twice as large as those based on *F*, and *R*- factors based on ALL data will be even larger. H-atoms attached to carbon were placed in calculated positions (C—H = 0.95 - 0.98 Å) while those attached to nitrogen were placed in locations derived from a difference map and their parameters adjusted to give N—H = 0.91 Å. All were included as riding contributions with isotropic displacement parameters 1.2 - 1.5 times those of the attached atoms. The major portion of the side chain in molecule 1 is disordered over two reasonably resolved sites in a 4:1 ratio. The two components of the disorder were refined subject to restraints that their geometries be comparable to one another and to that of the corresponding ordered portion of molecule 2.

### Fractional atomic coordinates and isotropic or equivalent isotropic displacement parameters (Å^2^)

**Table:**

	*x*	*y*	*z*	*U*_iso_*/*U*_eq_	Occ. (<1)
O1	0.6238 (3)	0.3465 (3)	0.28680 (16)	0.0306 (5)	0.799 (2)
O2	0.77612 (17)	0.15788 (14)	0.22373 (8)	0.0302 (3)	0.799 (2)
C12	0.5735 (15)	0.2907 (8)	0.1590 (4)	0.0254 (15)	0.799 (2)
C13	0.6233 (9)	0.1924 (11)	0.0963 (6)	0.0257 (11)	0.799 (2)
C14	0.6579 (3)	0.2695 (2)	0.22932 (15)	0.0253 (5)	0.799 (2)
C15	0.8661 (3)	0.1257 (2)	0.29189 (13)	0.0320 (5)	0.799 (2)
H15A	0.8749	0.2093	0.3140	0.038*	0.799 (2)
H15B	0.9722	0.0690	0.2727	0.038*	0.799 (2)
C16	0.7883 (3)	0.0527 (2)	0.35783 (14)	0.0395 (5)	0.799 (2)
H16A	0.8495	0.0315	0.4034	0.059*	0.799 (2)
H16B	0.6838	0.1096	0.3772	0.059*	0.799 (2)
H16C	0.7808	−0.0305	0.3359	0.059*	0.799 (2)
O1A	0.5872 (14)	0.3347 (15)	0.2989 (8)	0.0306 (5)	0.201 (2)
O2A	0.6939 (7)	0.1234 (6)	0.2493 (3)	0.0302 (3)	0.201 (2)
C12A	0.562 (7)	0.284 (3)	0.164 (2)	0.0254 (15)	0.201 (2)
C13A	0.601 (4)	0.190 (5)	0.098 (3)	0.0257 (11)	0.201 (2)
C14A	0.6162 (13)	0.2541 (11)	0.2439 (7)	0.0253 (5)	0.201 (2)
C15A	0.7531 (10)	0.0775 (9)	0.3268 (5)	0.0320 (5)	0.201 (2)
H15C	0.7664	−0.0200	0.3337	0.038*	0.201 (2)
H15D	0.6767	0.1237	0.3726	0.038*	0.201 (2)
C16A	0.9041 (11)	0.1054 (10)	0.3289 (6)	0.0395 (5)	0.201 (2)
H16D	0.9413	0.0740	0.3812	0.059*	0.201 (2)
H16E	0.9802	0.0585	0.2841	0.059*	0.201 (2)
H16F	0.8906	0.2021	0.3230	0.059*	0.201 (2)
N1	0.23080 (14)	0.71344 (12)	0.25293 (7)	0.0246 (3)	
N2	0.12067 (15)	0.82212 (13)	0.22657 (8)	0.0272 (3)	
N3	0.40300 (14)	0.50200 (12)	0.20513 (7)	0.0250 (3)	
H3A	0.4491	0.4869	0.2513	0.030*	
N4	0.65730 (18)	0.11313 (15)	0.04604 (9)	0.0373 (3)	
C1	0.25120 (18)	0.70254 (15)	0.33706 (9)	0.0239 (3)	
C2	0.12130 (19)	0.73850 (17)	0.39628 (10)	0.0303 (4)	
H2	0.0203	0.7707	0.3807	0.036*	
C3	0.1403 (2)	0.72708 (18)	0.47835 (10)	0.0354 (4)	
H3	0.0518	0.7520	0.5189	0.042*	
C4	0.2871 (2)	0.67965 (17)	0.50155 (10)	0.0327 (4)	
H4	0.2995	0.6708	0.5577	0.039*	
C5	0.41506 (19)	0.64536 (16)	0.44225 (10)	0.0306 (4)	
H5	0.5159	0.6127	0.4581	0.037*	
C6	0.39891 (18)	0.65784 (16)	0.35969 (9)	0.0267 (3)	
H6	0.4880	0.6360	0.3193	0.032*	
C7	0.11687 (18)	0.79590 (16)	0.14906 (9)	0.0273 (3)	
C8	0.22272 (18)	0.67160 (16)	0.12446 (9)	0.0272 (3)	
H8	0.2418	0.6312	0.0724	0.033*	
C9	0.29172 (17)	0.62220 (15)	0.19198 (9)	0.0233 (3)	
C10	0.0078 (2)	0.89208 (17)	0.09971 (10)	0.0347 (4)	
H10A	−0.0727	0.8522	0.0886	0.052*	
H10B	0.0654	0.9119	0.0480	0.052*	
H10C	−0.0409	0.9752	0.1302	0.052*	
C11	0.45577 (17)	0.40469 (15)	0.14963 (9)	0.0251 (3)	
H11	0.4090	0.4152	0.1011	0.030*	
O3	0.66182 (14)	0.82942 (11)	0.26058 (7)	0.0354 (3)	
O4	0.79855 (13)	0.63165 (11)	0.20236 (7)	0.0302 (3)	
N5	0.23310 (15)	1.18094 (13)	0.23448 (8)	0.0287 (3)	
N6	0.12144 (16)	1.28758 (13)	0.20738 (8)	0.0314 (3)	
N7	0.42346 (15)	0.97824 (13)	0.18512 (8)	0.0288 (3)	
H7A	0.4804	0.9711	0.2269	0.035*	
N8	0.65249 (17)	0.57821 (15)	0.03377 (9)	0.0369 (3)	
C17	0.26044 (18)	1.17753 (16)	0.31716 (10)	0.0288 (3)	
C18	0.2914 (2)	1.05786 (17)	0.36117 (10)	0.0363 (4)	
H18	0.2932	0.9768	0.3367	0.044*	
C19	0.3195 (2)	1.05798 (19)	0.44122 (11)	0.0428 (4)	
H19	0.3444	0.9758	0.4710	0.051*	
C20	0.3119 (2)	1.1756 (2)	0.47826 (11)	0.0428 (4)	
H20	0.3308	1.1746	0.5333	0.051*	
C21	0.2767 (2)	1.29494 (19)	0.43479 (11)	0.0401 (4)	
H21	0.2695	1.3765	0.4604	0.048*	
C22	0.2519 (2)	1.29647 (17)	0.35414 (10)	0.0332 (4)	
H22	0.2292	1.3786	0.3243	0.040*	
C23	0.12004 (19)	1.26028 (17)	0.12999 (10)	0.0323 (4)	
C24	0.2316 (2)	1.13890 (17)	0.10562 (10)	0.0335 (4)	
H24	0.2537	1.0985	0.0534	0.040*	
C25	0.30107 (18)	1.09202 (16)	0.17336 (10)	0.0288 (3)	
C26	0.0069 (2)	1.35258 (19)	0.08089 (11)	0.0432 (5)	
H26A	−0.0709	1.3090	0.0704	0.065*	
H26B	0.0623	1.3738	0.0288	0.065*	
H26C	−0.0448	1.4354	0.1112	0.065*	
C27	0.46945 (18)	0.87682 (16)	0.13317 (9)	0.0283 (3)	
H27	0.4135	0.8819	0.0883	0.034*	
C28	0.59263 (18)	0.76496 (16)	0.13985 (9)	0.0270 (3)	
C29	0.62621 (18)	0.66170 (16)	0.08068 (9)	0.0282 (3)	
C30	0.68579 (18)	0.74789 (15)	0.20653 (9)	0.0269 (3)	
C31	0.90350 (19)	0.60631 (17)	0.26367 (10)	0.0316 (4)	
H31A	0.9230	0.6914	0.2763	0.038*	
H31B	1.0041	0.5437	0.2414	0.038*	
C32	0.8387 (2)	0.5478 (2)	0.34076 (11)	0.0393 (4)	
H32A	0.7475	0.6149	0.3674	0.059*	
H32B	0.9178	0.5214	0.3776	0.059*	
H32C	0.8084	0.4690	0.3277	0.059*	

### Atomic displacement parameters (Å^2^)

**Table:**

	*U*^11^	*U*^22^	*U*^33^	*U*^12^	*U*^13^	*U*^23^
O1	0.0297 (15)	0.0298 (9)	0.0285 (11)	0.0012 (9)	−0.0088 (9)	−0.0081 (8)
O2	0.0315 (8)	0.0278 (7)	0.0259 (7)	0.0031 (6)	−0.0076 (6)	−0.0044 (6)
C12	0.028 (2)	0.0240 (12)	0.0232 (12)	−0.0052 (15)	−0.0032 (16)	−0.0026 (10)
C13	0.023 (3)	0.0275 (9)	0.0246 (9)	−0.0021 (19)	−0.0060 (17)	−0.0007 (8)
C14	0.0240 (14)	0.0240 (10)	0.0253 (12)	−0.0021 (9)	−0.0034 (9)	−0.0024 (8)
C15	0.0330 (11)	0.0298 (11)	0.0294 (11)	0.0014 (8)	−0.0125 (9)	−0.0019 (8)
C16	0.0514 (14)	0.0342 (12)	0.0337 (12)	−0.0091 (10)	−0.0145 (10)	0.0000 (10)
O1A	0.0297 (15)	0.0298 (9)	0.0285 (11)	0.0012 (9)	−0.0088 (9)	−0.0081 (8)
O2A	0.0315 (8)	0.0278 (7)	0.0259 (7)	0.0031 (6)	−0.0076 (6)	−0.0044 (6)
C12A	0.028 (2)	0.0240 (12)	0.0232 (12)	−0.0052 (15)	−0.0032 (16)	−0.0026 (10)
C13A	0.023 (3)	0.0275 (9)	0.0246 (9)	−0.0021 (19)	−0.0060 (17)	−0.0007 (8)
C14A	0.0240 (14)	0.0240 (10)	0.0253 (12)	−0.0021 (9)	−0.0034 (9)	−0.0024 (8)
C15A	0.0330 (11)	0.0298 (11)	0.0294 (11)	0.0014 (8)	−0.0125 (9)	−0.0019 (8)
C16A	0.0514 (14)	0.0342 (12)	0.0337 (12)	−0.0091 (10)	−0.0145 (10)	0.0000 (10)
N1	0.0252 (6)	0.0237 (7)	0.0227 (6)	−0.0019 (5)	−0.0060 (5)	−0.0011 (5)
N2	0.0274 (7)	0.0245 (7)	0.0265 (7)	−0.0005 (5)	−0.0070 (5)	−0.0001 (5)
N3	0.0262 (7)	0.0241 (7)	0.0218 (6)	−0.0009 (5)	−0.0062 (5)	−0.0011 (5)
N4	0.0416 (8)	0.0345 (8)	0.0298 (7)	0.0021 (7)	−0.0089 (6)	−0.0074 (6)
C1	0.0289 (8)	0.0211 (7)	0.0223 (7)	−0.0064 (6)	−0.0060 (6)	−0.0021 (6)
C2	0.0259 (8)	0.0344 (9)	0.0294 (8)	−0.0050 (7)	−0.0054 (6)	−0.0062 (7)
C3	0.0353 (9)	0.0422 (10)	0.0268 (8)	−0.0090 (8)	0.0007 (7)	−0.0092 (7)
C4	0.0429 (10)	0.0343 (9)	0.0232 (8)	−0.0120 (8)	−0.0085 (7)	−0.0020 (7)
C5	0.0322 (9)	0.0306 (9)	0.0304 (8)	−0.0071 (7)	−0.0122 (7)	−0.0007 (7)
C6	0.0262 (8)	0.0279 (8)	0.0258 (8)	−0.0070 (6)	−0.0036 (6)	−0.0021 (6)
C7	0.0274 (8)	0.0273 (8)	0.0256 (8)	−0.0049 (7)	−0.0051 (6)	0.0009 (6)
C8	0.0290 (8)	0.0283 (8)	0.0218 (7)	−0.0030 (6)	−0.0052 (6)	−0.0010 (6)
C9	0.0229 (7)	0.0232 (7)	0.0227 (7)	−0.0046 (6)	−0.0039 (6)	−0.0004 (6)
C10	0.0349 (9)	0.0357 (9)	0.0281 (8)	0.0016 (7)	−0.0102 (7)	0.0005 (7)
C11	0.0270 (8)	0.0264 (8)	0.0210 (7)	−0.0055 (6)	−0.0046 (6)	−0.0011 (6)
O3	0.0399 (7)	0.0287 (6)	0.0363 (6)	−0.0034 (5)	−0.0105 (5)	−0.0090 (5)
O4	0.0302 (6)	0.0273 (6)	0.0307 (6)	−0.0009 (5)	−0.0093 (5)	−0.0044 (5)
N5	0.0287 (7)	0.0239 (7)	0.0306 (7)	−0.0016 (5)	−0.0060 (5)	−0.0008 (5)
N6	0.0325 (7)	0.0247 (7)	0.0331 (7)	0.0001 (6)	−0.0080 (6)	−0.0003 (6)
N7	0.0300 (7)	0.0263 (7)	0.0283 (7)	−0.0026 (6)	−0.0080 (5)	−0.0025 (5)
N8	0.0409 (8)	0.0357 (8)	0.0303 (7)	−0.0013 (7)	−0.0104 (6)	−0.0047 (6)
C17	0.0250 (8)	0.0306 (8)	0.0287 (8)	−0.0045 (7)	−0.0034 (6)	−0.0006 (6)
C18	0.0410 (10)	0.0279 (9)	0.0351 (9)	−0.0033 (7)	−0.0024 (7)	−0.0005 (7)
C19	0.0467 (11)	0.0398 (10)	0.0340 (9)	−0.0011 (8)	−0.0051 (8)	0.0067 (8)
C20	0.0415 (10)	0.0523 (12)	0.0329 (9)	−0.0070 (9)	−0.0116 (8)	−0.0010 (8)
C21	0.0440 (10)	0.0416 (10)	0.0382 (10)	−0.0143 (8)	−0.0106 (8)	−0.0048 (8)
C22	0.0355 (9)	0.0308 (9)	0.0346 (9)	−0.0103 (7)	−0.0073 (7)	0.0005 (7)
C23	0.0318 (9)	0.0285 (9)	0.0337 (9)	−0.0030 (7)	−0.0061 (7)	−0.0017 (7)
C24	0.0353 (9)	0.0306 (9)	0.0323 (9)	−0.0043 (7)	−0.0051 (7)	−0.0050 (7)
C25	0.0279 (8)	0.0241 (8)	0.0327 (8)	−0.0043 (6)	−0.0045 (6)	−0.0016 (6)
C26	0.0433 (10)	0.0405 (10)	0.0377 (10)	0.0051 (8)	−0.0126 (8)	−0.0026 (8)
C27	0.0293 (8)	0.0293 (8)	0.0258 (8)	−0.0067 (7)	−0.0054 (6)	−0.0011 (6)
C28	0.0273 (8)	0.0258 (8)	0.0267 (8)	−0.0058 (6)	−0.0039 (6)	0.0000 (6)
C29	0.0249 (8)	0.0289 (8)	0.0273 (8)	−0.0015 (6)	−0.0061 (6)	0.0029 (7)
C30	0.0277 (8)	0.0219 (8)	0.0296 (8)	−0.0059 (6)	−0.0011 (6)	−0.0021 (6)
C31	0.0281 (8)	0.0347 (9)	0.0327 (8)	−0.0063 (7)	−0.0111 (7)	−0.0016 (7)
C32	0.0380 (10)	0.0470 (11)	0.0352 (9)	−0.0134 (8)	−0.0106 (8)	0.0031 (8)

### Geometric parameters (Å, º)

**Table:**

O1—C14	1.223 (3)	C8—C9	1.366 (2)
O2—C14	1.333 (3)	C8—H8	0.9500
O2—C15	1.457 (2)	C10—H10A	0.9800
C12—C11	1.370 (5)	C10—H10B	0.9800
C12—C13	1.428 (4)	C10—H10C	0.9800
C12—C14	1.458 (5)	C11—H11	0.9500
C13—N4	1.149 (4)	O3—C30	1.2186 (18)
C15—C16	1.503 (3)	O4—C30	1.3396 (19)
C15—H15A	0.9900	O4—C31	1.4550 (18)
C15—H15B	0.9900	N5—C25	1.360 (2)
C16—H16A	0.9800	N5—N6	1.3772 (18)
C16—H16B	0.9800	N5—C17	1.423 (2)
C16—H16C	0.9800	N6—C23	1.329 (2)
O1A—C14A	1.218 (13)	N7—C27	1.329 (2)
O2A—C14A	1.346 (12)	N7—C25	1.400 (2)
O2A—C15A	1.467 (9)	N7—H7A	0.9100
C12A—C11	1.379 (15)	N8—C29	1.146 (2)
C12A—C13A	1.442 (15)	C17—C22	1.387 (2)
C12A—C14A	1.473 (15)	C17—C18	1.388 (2)
C13A—N4	1.157 (15)	C18—C19	1.386 (2)
C15A—C16A	1.484 (11)	C18—H18	0.9500
C15A—H15C	0.9900	C19—C20	1.377 (3)
C15A—H15D	0.9900	C19—H19	0.9500
C16A—H16D	0.9800	C20—C21	1.382 (3)
C16A—H16E	0.9800	C20—H20	0.9500
C16A—H16F	0.9800	C21—C22	1.385 (2)
N1—C9	1.3648 (19)	C21—H21	0.9500
N1—N2	1.3773 (17)	C22—H22	0.9500
N1—C1	1.4261 (18)	C23—C24	1.409 (2)
N2—C7	1.3331 (19)	C23—C26	1.495 (2)
N3—C11	1.3382 (19)	C24—C25	1.366 (2)
N3—C9	1.3945 (19)	C24—H24	0.9500
N3—H3A	0.9099	C26—H26A	0.9800
C1—C6	1.387 (2)	C26—H26B	0.9800
C1—C2	1.391 (2)	C26—H26C	0.9800
C2—C3	1.389 (2)	C27—C28	1.376 (2)
C2—H2	0.9500	C27—H27	0.9500
C3—C4	1.384 (2)	C28—C29	1.429 (2)
C3—H3	0.9500	C28—C30	1.460 (2)
C4—C5	1.380 (2)	C31—C32	1.499 (2)
C4—H4	0.9500	C31—H31A	0.9900
C5—C6	1.391 (2)	C31—H31B	0.9900
C5—H5	0.9500	C32—H32A	0.9800
C6—H6	0.9500	C32—H32B	0.9800
C7—C8	1.411 (2)	C32—H32C	0.9800
C7—C10	1.494 (2)		
			
C14—O2—C15	117.05 (16)	C7—C10—H10A	109.5
C11—C12—C13	119.8 (6)	C7—C10—H10B	109.5
C11—C12—C14	122.0 (4)	H10A—C10—H10B	109.5
C13—C12—C14	118.1 (6)	C7—C10—H10C	109.5
N4—C13—C12	177.2 (9)	H10A—C10—H10C	109.5
O1—C14—O2	124.0 (3)	H10B—C10—H10C	109.5
O1—C14—C12	123.4 (3)	N3—C11—C12	123.6 (2)
O2—C14—C12	112.7 (2)	N3—C11—C12A	123.0 (7)
O2—C15—C16	110.05 (19)	N3—C11—H11	118.2
O2—C15—H15A	109.7	C12—C11—H11	118.2
C16—C15—H15A	109.7	C30—O4—C31	117.32 (12)
O2—C15—H15B	109.7	C25—N5—N6	110.68 (13)
C16—C15—H15B	109.7	C25—N5—C17	130.18 (13)
H15A—C15—H15B	108.2	N6—N5—C17	119.14 (13)
C15—C16—H16A	109.5	C23—N6—N5	104.99 (13)
C15—C16—H16B	109.5	C27—N7—C25	121.93 (13)
H16A—C16—H16B	109.5	C27—N7—H7A	115.7
C15—C16—H16C	109.5	C25—N7—H7A	122.3
H16A—C16—H16C	109.5	C22—C17—C18	120.14 (15)
H16B—C16—H16C	109.5	C22—C17—N5	119.02 (15)
C14A—O2A—C15A	116.5 (7)	C18—C17—N5	120.82 (15)
C11—C12A—C13A	115 (3)	C19—C18—C17	119.25 (16)
C11—C12A—C14A	120.2 (13)	C19—C18—H18	120.4
C13A—C12A—C14A	125 (3)	C17—C18—H18	120.4
N4—C13A—C12A	168 (4)	C20—C19—C18	120.91 (17)
O1A—C14A—O2A	125.2 (13)	C20—C19—H19	119.5
O1A—C14A—C12A	124.6 (14)	C18—C19—H19	119.5
O2A—C14A—C12A	110.1 (11)	C19—C20—C21	119.51 (17)
O2A—C15A—C16A	111.0 (8)	C19—C20—H20	120.2
O2A—C15A—H15C	109.4	C21—C20—H20	120.2
C16A—C15A—H15C	109.4	C20—C21—C22	120.45 (17)
O2A—C15A—H15D	109.4	C20—C21—H21	119.8
C16A—C15A—H15D	109.4	C22—C21—H21	119.8
H15C—C15A—H15D	108.0	C21—C22—C17	119.69 (16)
C15A—C16A—H16D	109.5	C21—C22—H22	120.2
C15A—C16A—H16E	109.5	C17—C22—H22	120.2
H16D—C16A—H16E	109.5	N6—C23—C24	111.52 (15)
C15A—C16A—H16F	109.5	N6—C23—C26	120.31 (15)
H16D—C16A—H16F	109.5	C24—C23—C26	128.16 (16)
H16E—C16A—H16F	109.5	C25—C24—C23	104.98 (15)
C9—N1—N2	110.60 (12)	C25—C24—H24	127.5
C9—N1—C1	129.64 (12)	C23—C24—H24	127.5
N2—N1—C1	119.23 (12)	N5—C25—C24	107.81 (14)
C7—N2—N1	104.92 (12)	N5—C25—N7	121.23 (14)
C11—N3—C9	122.93 (13)	C24—C25—N7	130.93 (15)
C11—N3—H3A	115.4	C23—C26—H26A	109.5
C9—N3—H3A	121.6	C23—C26—H26B	109.5
C6—C1—C2	120.28 (14)	H26A—C26—H26B	109.5
C6—C1—N1	120.43 (14)	C23—C26—H26C	109.5
C2—C1—N1	119.29 (14)	H26A—C26—H26C	109.5
C3—C2—C1	119.57 (15)	H26B—C26—H26C	109.5
C3—C2—H2	120.2	N7—C27—C28	124.50 (14)
C1—C2—H2	120.2	N7—C27—H27	117.8
C4—C3—C2	120.55 (15)	C28—C27—H27	117.8
C4—C3—H3	119.7	C27—C28—C29	118.43 (14)
C2—C3—H3	119.7	C27—C28—C30	121.86 (14)
C5—C4—C3	119.33 (15)	C29—C28—C30	119.69 (14)
C5—C4—H4	120.3	N8—C29—C28	179.35 (19)
C3—C4—H4	120.3	O3—C30—O4	123.93 (15)
C4—C5—C6	121.09 (15)	O3—C30—C28	123.66 (14)
C4—C5—H5	119.5	O4—C30—C28	112.41 (13)
C6—C5—H5	119.5	O4—C31—C32	111.97 (14)
C1—C6—C5	119.16 (15)	O4—C31—H31A	109.2
C1—C6—H6	120.4	C32—C31—H31A	109.2
C5—C6—H6	120.4	O4—C31—H31B	109.2
N2—C7—C8	111.68 (13)	C32—C31—H31B	109.2
N2—C7—C10	120.50 (14)	H31A—C31—H31B	107.9
C8—C7—C10	127.81 (14)	C31—C32—H32A	109.5
C9—C8—C7	104.81 (13)	C31—C32—H32B	109.5
C9—C8—H8	127.6	H32A—C32—H32B	109.5
C7—C8—H8	127.6	C31—C32—H32C	109.5
N1—C9—C8	107.99 (13)	H32A—C32—H32C	109.5
N1—C9—N3	120.92 (13)	H32B—C32—H32C	109.5
C8—C9—N3	131.09 (14)		
			
C15—O2—C14—C12	−178.7 (7)	C13—C12—C11—N3	−179.5 (9)
C11—C12—C14—O1	4.9 (17)	C14—C12—C11—N3	−3.5 (17)
C13—C12—C14—O1	−179.1 (9)	C13—C12—C11—C12A	94 (10)
C11—C12—C14—O2	−174.8 (10)	C14—C12—C11—C12A	−90 (9)
C13—C12—C14—O2	1.2 (15)	C13A—C12A—C11—N3	178 (4)
C14—O2—C15—C16	84.3 (2)	C14A—C12A—C11—N3	4 (8)
C11—C12A—C13A—N4	128 (28)	C13A—C12A—C11—C12	−85 (9)
C14A—C12A—C13A—N4	−58 (33)	C25—N5—N6—C23	1.57 (18)
C15A—O2A—C14A—O1A	−2.7 (15)	C17—N5—N6—C23	−178.15 (14)
C15A—O2A—C14A—C12A	−179 (3)	C25—N5—C17—C22	143.45 (17)
C11—C12A—C14A—O1A	−8 (8)	N6—N5—C17—C22	−36.9 (2)
C13A—C12A—C14A—O1A	178 (4)	C25—N5—C17—C18	−38.2 (3)
C11—C12A—C14A—O2A	168 (4)	N6—N5—C17—C18	141.49 (16)
C13A—C12A—C14A—O2A	−6 (7)	C22—C17—C18—C19	−2.5 (3)
C14A—O2A—C15A—C16A	−83.1 (10)	N5—C17—C18—C19	179.13 (16)
C9—N1—N2—C7	−0.50 (17)	C17—C18—C19—C20	2.2 (3)
C1—N1—N2—C7	−172.92 (13)	C18—C19—C20—C21	−0.4 (3)
C12A—C13A—N4—C13	−15 (13)	C19—C20—C21—C22	−1.2 (3)
C9—N1—C1—C6	49.5 (2)	C20—C21—C22—C17	0.9 (3)
N2—N1—C1—C6	−139.71 (15)	C18—C17—C22—C21	1.0 (3)
C9—N1—C1—C2	−131.12 (17)	N5—C17—C22—C21	179.36 (15)
N2—N1—C1—C2	39.7 (2)	N5—N6—C23—C24	−1.36 (19)
C6—C1—C2—C3	−1.2 (2)	N5—N6—C23—C26	177.54 (16)
N1—C1—C2—C3	179.48 (15)	N6—C23—C24—C25	0.7 (2)
C1—C2—C3—C4	−0.3 (3)	C26—C23—C24—C25	−178.12 (18)
C2—C3—C4—C5	0.9 (3)	N6—N5—C25—C24	−1.20 (19)
C3—C4—C5—C6	0.1 (3)	C17—N5—C25—C24	178.49 (16)
C2—C1—C6—C5	2.1 (2)	N6—N5—C25—N7	176.97 (14)
N1—C1—C6—C5	−178.57 (14)	C17—N5—C25—N7	−3.3 (3)
C4—C5—C6—C1	−1.5 (2)	C23—C24—C25—N5	0.33 (19)
N1—N2—C7—C8	0.09 (18)	C23—C24—C25—N7	−177.59 (17)
N1—N2—C7—C10	179.26 (14)	C27—N7—C25—N5	165.61 (15)
N2—C7—C8—C9	0.33 (19)	C27—N7—C25—C24	−16.7 (3)
C10—C7—C8—C9	−178.75 (16)	C25—N7—C27—C28	177.38 (15)
N2—N1—C9—C8	0.72 (17)	N7—C27—C28—C29	178.21 (15)
C1—N1—C9—C8	172.13 (15)	N7—C27—C28—C30	0.0 (3)
N2—N1—C9—N3	−178.38 (13)	C31—O4—C30—O3	−3.8 (2)
C1—N1—C9—N3	−7.0 (2)	C31—O4—C30—C28	177.29 (13)
C7—C8—C9—N1	−0.62 (17)	C27—C28—C30—O3	0.3 (3)
C7—C8—C9—N3	178.36 (16)	C29—C28—C30—O3	−177.87 (15)
C11—N3—C9—N1	173.94 (14)	C27—C28—C30—O4	179.22 (14)
C11—N3—C9—C8	−4.9 (3)	C29—C28—C30—O4	1.0 (2)
C9—N3—C11—C12	174.7 (9)	C30—O4—C31—C32	84.67 (18)
C9—N3—C11—C12A	−178 (4)		

### Hydrogen-bond geometry (Å, º)

**Table:**

*D*—H···*A*	*D*—H	H···*A*	*D*···*A*	*D*—H···*A*
C15—H15*B*···N2^i^	0.99	2.63	3.455 (2)	141
C16—H16*C*···O3^ii^	0.98	2.52	3.430 (3)	155
N3—H3*A*···O1	0.91	1.96	2.677 (4)	134
C10—H10*C*···O2^iii^	0.98	2.55	3.506 (2)	164
C11—H11···N8^iv^	0.95	2.37	3.306 (2)	168
N7—H7*A*···O3	0.91	2.00	2.7027 (17)	133
C24—H24···N4^iv^	0.95	2.68	3.555 (2)	153
C26—H26*C*···O4^iii^	0.98	2.55	3.523 (2)	172
C27—H27···N4^iv^	0.95	2.40	3.322 (2)	164
C31—H31*A*···N2^v^	0.99	2.57	3.366 (2)	138
C31—H31*B*···N6^i^	0.99	2.63	3.437 (2)	139
C32—H32*C*···O1	0.98	2.55	3.468 (3)	155

Symmetry codes: (i) *x*+1, *y*−1, *z*; (ii) *x*, *y*−1, *z*; (iii) *x*−1, *y*+1, *z*; (iv) −*x*+1, −*y*+1, −*z*; (v) *x*+1, *y*, *z*.
