# Supplementary figures and images for: Crystal structure of ethyl (2Z)-2-cyano-3-[(3-methyl-1-phenyl-1H-pyrazol-5-yl)amino]­prop-2-enoate
Source: Acta Crystallogr Sect E Struct Rep Online. 2014 Oct 31;70(Pt 11):o1214–5. doi: 10.1107/S1600536814023502 (PMC4257344; doi:10.1107/S1600536814023502)

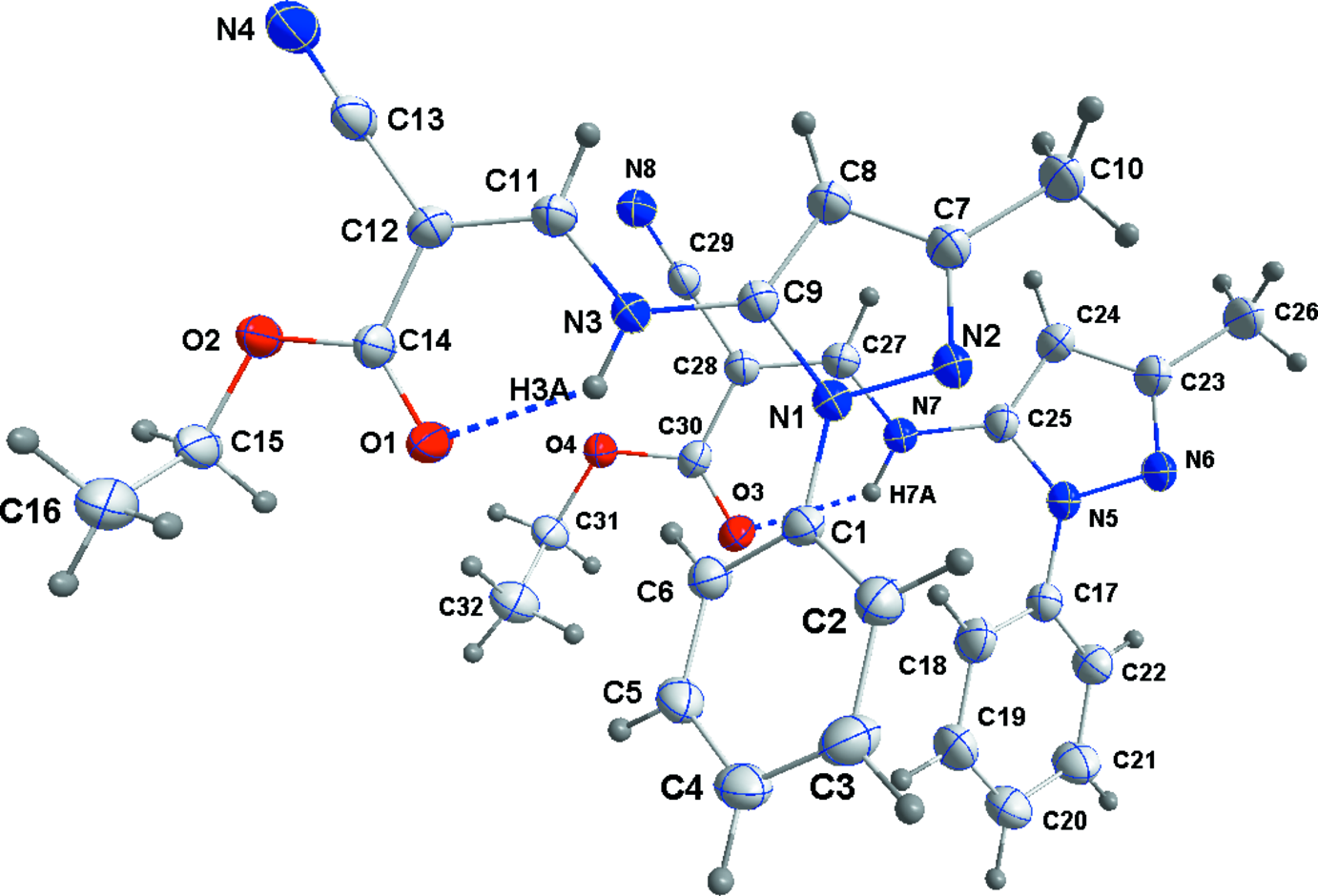

Supplement: Supplementary file 4 [file e-70-o1214-fig1.tif]

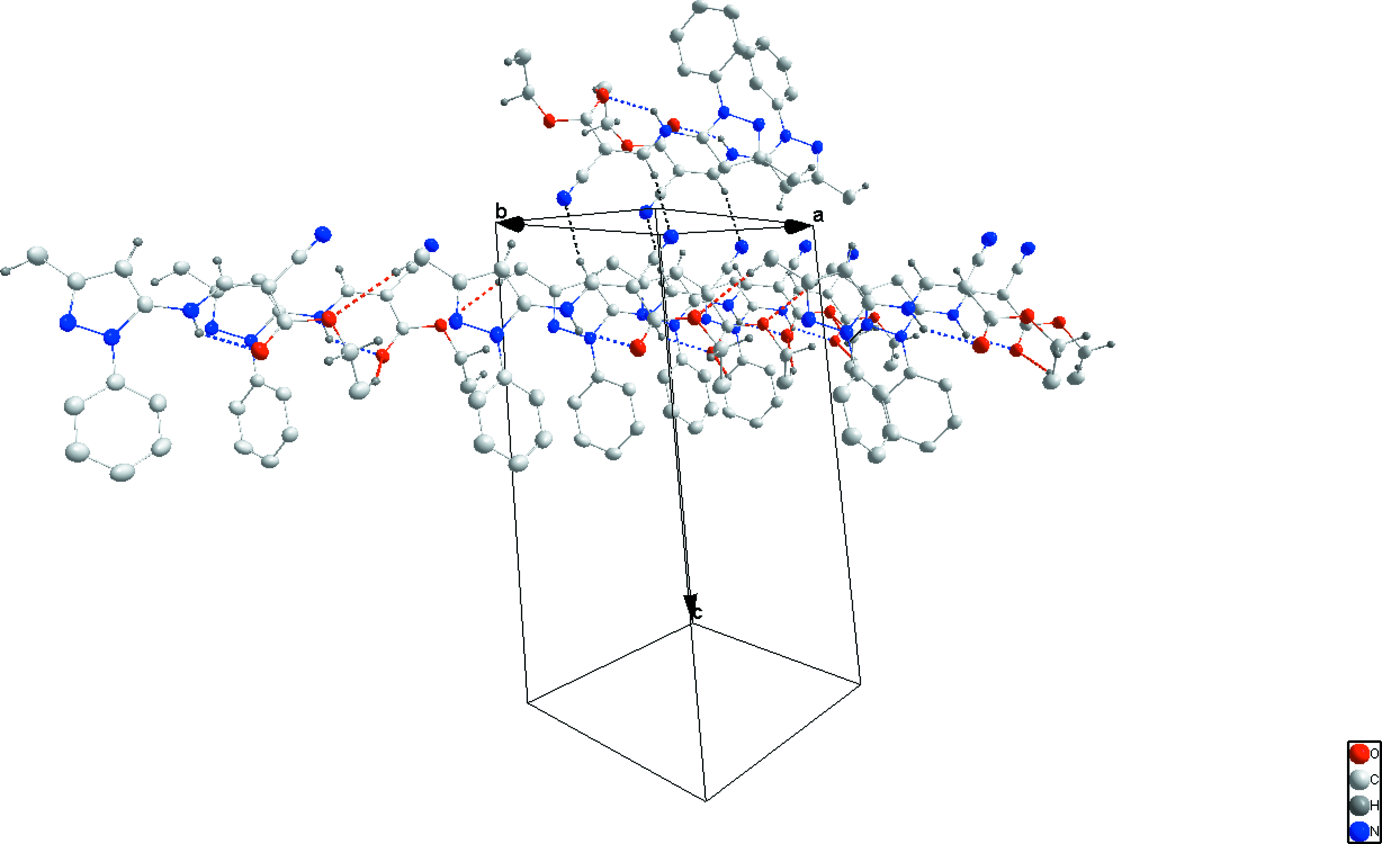

Supplement: Supplementary file 5 [file e-70-o1214-fig2.tif]

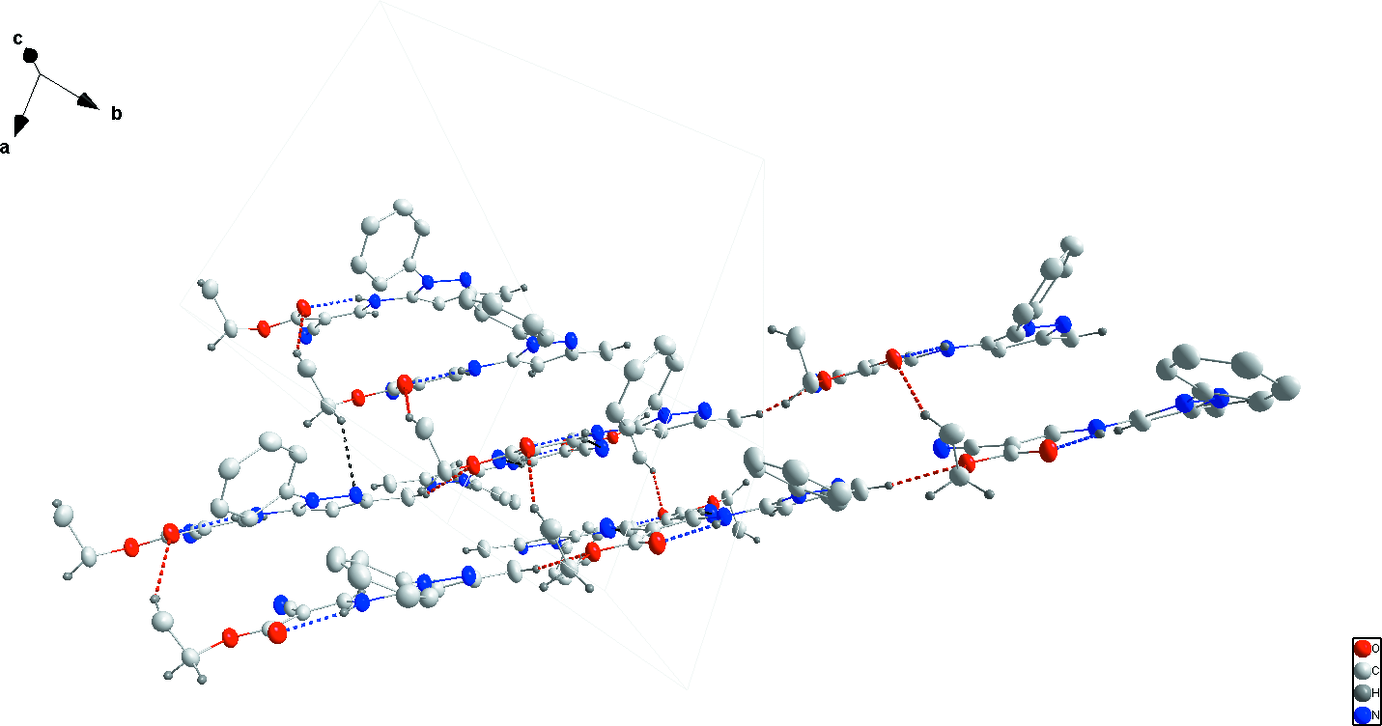

Supplement: Supplementary file 6 [file e-70-o1214-fig3.tif]
